# Split-marker-mediated genome editing improves homologous recombination frequency in the CTG clade yeast *Candida intermedia*

**DOI:** 10.1093/femsyr/foad016

**Published:** 2023-03-09

**Authors:** Kameshwara V R Peri, Fábio Faria-Oliveira, Adam Larsson, Alexander Plovie, Nicolas Papon, Cecilia Geijer

**Affiliations:** Chalmers University of Technology, Department of Life Sciences, Division of Industrial Biotechnology, Kemigården 1, 41296 Gothenburg, Sweden; Chalmers University of Technology, Department of Life Sciences, Division of Industrial Biotechnology, Kemigården 1, 41296 Gothenburg, Sweden; Chalmers University of Technology, Department of Life Sciences, Division of Industrial Biotechnology, Kemigården 1, 41296 Gothenburg, Sweden; Chalmers University of Technology, Department of Life Sciences, Division of Industrial Biotechnology, Kemigården 1, 41296 Gothenburg, Sweden; University of Angers, Univ Brest, IRF, SFR ICAT, F-49000 Angers, France; Chalmers University of Technology, Department of Life Sciences, Division of Industrial Biotechnology, Kemigården 1, 41296 Gothenburg, Sweden

**Keywords:** nonhomologous end-joining, nonconventional yeast, cell factory development

## Abstract

Genome-editing toolboxes are essential for the exploration and exploitation of nonconventional yeast species as cell factories, as they facilitate both genome studies and metabolic engineering. The nonconventional yeast *Candida intermedia* is a biotechnologically interesting species due to its capacity to convert a wide range of carbon sources, including xylose and lactose found in forestry and dairy industry waste and side-streams, into added-value products. However, possibilities of genetic manipulation have so far been limited due to lack of molecular tools for this species. We describe here the development of a genome editing method for *C. intermedia*, based on electroporation and gene deletion cassettes containing the *Candida albicans NAT1* dominant selection marker flanked by 1000 base pair sequences homologous to the target loci. Linear deletion cassettes targeting the *ADE2* gene originally resulted in <1% targeting efficiencies, suggesting that *C. intermedia* mainly uses nonhomologous end joining for integration of foreign DNA fragments. By developing a split-marker based deletion technique for *C. intermedia*, we successfully improved the homologous recombination rates, achieving targeting efficiencies up to 70%. For marker-less deletions, we also employed the split-marker cassette in combination with a recombinase system, which enabled the construction of double deletion mutants via marker recycling. Overall, the split-marker technique proved to be a quick and reliable method for generating gene deletions in *C. intermedia*, which opens the possibility to uncover and enhance its cell factory potential.

## Teaser

This article describes the development of genome editing tools for the CTG clade yeast *Candida intermedia*.

## Introduction

A biobased society relies on efficient utilization of renewable raw materials for production of fuels, chemicals, and materials. In this regard, microbial cell factories that can convert various carbon sources found in industrial waste and side-streams into valuable products are highly sought after as key enablers of sustainable and circular production processes. Whereas the industrial workhorse *Saccharomyces cerevisiae* can only ferment a subset of monosaccharides, several so called nonconventional yeast species display broad substrate ranges, often including various monosaccharides as well as disaccharides and sometimes even more complex oligo- and polysaccharides (Ramírez-Orozco et al. 2001, Lobs et al. 2017, Baptista and Domingues 2022, Šuchová et al. 2022). To develop a nonconventional yeast into a cell factory, detailed metabolic characterization on a molecular level remains an essential prerequisite. This typically requires genome editing tools that enable deletion of specific genes of interest and determination of genotype–phenotype relationships (Catlett et al. 2003). Moreover, targeted mutagenesis and expression of heterologous genes can further improve traits of a production host (Fonseca et al. 2011, Westfall et al. 2012).

*Candida intermedia* is a nonconventional yeast with an unusually broad substrate range (Shen et al. 2018). Its potential as a cell factory has been highlighted by the production of ethanol (Gardonyi et al. 2003, Fonseca et al. 2011, Geijer et al. 2020), xylitol, and single-cell protein (Wu et al. 2018) from xylose derived from lignocellulosic biomass. *Candida intermedia* has also been used for bioremediation of lactose found in the abundant industrial side stream cheese whey (Yonten and Aktas 2014), as well as production of antimicrobial peptides with application in wine preservation (Acuña-Fontecilla et al. 2017, Pena and Ganga 2019). We have previously *de novo* genome-sequenced the *C. intermedia* strain CBS 141442, which allows us to study the yeast at a genomic level (Geijer et al. 2020). Our analysis showed that *C. intermedia* is a haploid yeast belonging to the CTG clade of ascomycetous yeast, where the universal CUG codon is predominantly translated into a serine rather than a leucine (Geijer et al. 2020, Muhlhausen et al. 2021). The CTG clade comprises many relatively well-studied yeasts including human pathogens *Candida albicans, Candida tropicalis, Candida parapsilosis*, and industrially attractive yeasts such as *Debaryomyces hansenii, Metschnikowia pulcherrima*, and *Scheffersomyces stipitis* (Enkler et al. 2016, Cao et al. 2018, Muhlhausen et al. 2021, Strucko et al. 2021, Zhang et al. 2022). Genome editing tools have been developed for many of these species, which means that several CTG clade-compatible plasmids containing selectable marker genes and reporter systems are available (Papon et al. 2012, Lobs et al. 2017, Uthayakumar et al. 2021). These plasmids can be employed in the development of genetic engineering toolboxes for other yeast such as *C. intermedia*.

Genome editing often involves integration of a foreign DNA fragment in the host genome through the action of a double-strand break (DSB) repair mechanism, typically by nonhomologous end-joining (NHEJ) or homologous recombination (HR). The classical NHEJ mechanism is aided by the ku70/ku80 heterodimer and DNA ligase IV and involves error prone DSB repair where short (1–4 nucleotides) homologous fragments are used to repair the DSB. Ku-independent mechanisms are also known to exist, including microhomology-mediated end-joining (MMEJ) and single-strand annealing (SSA) that involve more extended homologies flanking the DSB (Wilson 2007). In contrast, HR involves DSB repair templated by the homologous sister chromosome, making it an error-free mechanism (Krejci et al. 2012, Sfeir and Symington 2015). Whereas NHEJ results in random integration of linear DNA fragments into the genome, HR targets DNA to a specific genomic locus (Orr-Weaver et al. 1981). NHEJ and HR coexist in most organisms but the balance in activity between them varies among species and cell types. *Saccharomyces cerevisiae* uses almost exclusively HR (Detloff et al. 1991) and as little as 20 bp genomic homology on each side of the selective marker gene is sufficient for targeted deletions (Corrigan et al. 2013). On the other hand, many members of the CTG clade seem to display a predominance of ectopic, NHEJ-mediated integrations (Lobs et al. 2017) necessitating long stretches of homology (0.5–2 kbp) to help direct the DNA fragment to the intended genome locus in many species (Nelson et al. 2003, Moreno-Beltran et al. 2021). Additionally, targeted gene deletions in species with low HR/NHEJ ratios typically require screening of many transformants to identify the desired mutant(s) (Fraczek et al. 2018).

Different approaches have been used to increase the HR frequency to make identification of mutants more efficient. Impairment of the NHEJ machinery by disrupting one of the genes encoding the ku70/ku80 heterodimer or other central NHEJ components has been shown to significantly increase HR in various yeast and fungal species (Kooistra et al. 2004, da Silva Ferreira et al. 2006, Haarmann et al. 2008, Kretzschmar et al. 2013). However, creating strains that are defective in the NHEJ pathway may be time consuming and labor intensive, and mutations in this pathway are known to compromise genome integrity maintenance (Barnes and Rio 1997, Dudášová et al. 2004, Kretzschmar et al. 2013, Raschmanová et al. 2018). Another approach to increase HR frequency, where NHEJ is left intact, is to benefit from the well-conserved cell cycle-dependent oscillation in HR/NHEJ preference of cells (Barnes and Rio 1997). Hydroxyurea treatment arrests cells in the S-phase of the cell cycle, where cells are more prone to use HR as sister chromatids are available as templates for repair. This way, targeting rates have been improved in yeast such as *Yarrowia lipolytica, S. cerevisiae, Kluyveromyces lactis*, and *Pichia pastoris* (Tsakraklides et al. 2015). A third way to promote HR is to use a split-marker transformation protocol, which was originally developed for rapid, gap repair-mediated, one step cloning in *S. cerevisiae*. Here, the homologous sequences of the target gene are fused to a selectable marker gene that is truncated in two different fragments with partial overlap in sequence, and only transformants in which HR between the overlapping parts has resulted in the constitution of a complete selection marker gene will be able to grow in selective medium. It has been shown that HR assembly of the marker fragments also promotes HR integration of the DNA cassette in the genome, resulting in a high frequency of targeted gene disruptions (Fairhead et al. 1996).

In many nonconventional yeasts, the limited availability of auxotrophic and antibiotic resistance markers is a real bottleneck for generation of multiple genome modifications in the same strain background. One solution is marker recycling, for example by making use of a flippase (Flp) adapted for CTG clade yeast (Morschhauser et al. 1999, Mancera et al. 2019). In this approach, the selectable marker is combined with the *FLP* gene and the whole cassette is flanked on either side by Flp recognition target (*FRT*) sequences. Flp catalyzes recombination of the *FRT* sequences and excision of the cassette, leaving only a small scar at the target locus. Another, more recently developed solution for markerless, multiple genome editing is the clustered regularly interspaced short palindromic repeat (CRISPR) and CRISPR-associated protein 9 (Cas9) (Jinek et al. 2012). Although the list of species that can be modified by this method expands every year, development of CRISPR/Cas9-based tools for new yeast species is not trivial and often requires species-specific optimization of gene expression, transformation, and selection protocols (Vyas et al. 2015, Ng and Dean 2017, Ennis et al. 2021, Strucko et al. 2021).

The aim of this study was to develop a first set of genome-editing tools for *C. intermedia*. The work included identification of a functional transformation protocol and deletion cassettes that enabled both single and double gene deletions in this yeast. Moreover, cell synchronization and in particular a split-marker approach improved the frequency of HR-mediated targeted genome integration of deletion cassettes, which may be useful also for future CRISPR/Cas9-based engineering of *C. intermedia* and other yeasts.

## Materials and methods

### Plasmids

#### *CaNAT1*-containing plasmids

Integrative plasmids p*CaNAT1* (pJK795) and p*FLP*_*CaNAT1* (pJK863) were provided by Dr. Julia Köhler, Boston Children’s Hospital, USA. Both plasmids are derivatives of plasmid pJK799 (Shen et al. 2005) and contain the *CaNAT1* selectable marker encoding nourseothricin acetyltransferase that has been shown to confer nourseothricin (cloNAT) resistance to *C. albicans* and other *Candida* species. The *CaNAT1* marker is expressed from the constitutive *TEF1* promoter and terminator in *Ashbya gossypii*. In addition to the *CaNAT1* marker, plasmid p*FLP*_*CaNAT1* contains a *FLP* gene encoding a FLP-recombinase driven by *C. albicans SAP2* promoter, which is induced by growing the yeast in medium with bovine serum albumin (BSA) as the sole nitrogen source (Morschhauser et al. 1999).

#### Construction of *ADE2* disruption plasmid

To generate the *ADE2* disruption cassette with *CaNAT1* marker, the *ADE2* gene from *C. intermedia* was PCR amplified along with 1000 bp upstream and downstream sequences and subsequently cloned into the multiple cloning site of a pBluescript II KS + vector using restriction enzymes KpnI and SacII and T4 DNA ligase (Thermo Fisher, Waltham, MA, USA) according to manufacturer’s instructions. The resulting plasmid was then digested using ClaI restriction enzyme (Thermo Fisher) that cuts in the middle of the *ADE2* gene. The *CaNAT1* marker, PCR-amplified from the p*CaNAT1* plasmid, was thereafter inserted at the cut site, creating plasmid p*ADE2disr_CaNAT1*.

#### Construction of additional gene deletion cassettes

Construction of deletion cassettes (including the *ADE2* deletion cassette with p*FLP_CaNAT1)* was done using molecular cloning: plasmids p*CaNAT1* or p*FLP_CaNAT1* were linearized and used as backbone for cloning the upstream and downstream homologous flanking region of the target gene next to the *CaNAT1* marker. Plasmid p*CaNAT1* was linearized using KpnI (for inserting an upstream homology sequence) or EcoRV (for inserting a downstream homology sequence) whereas p*FLP_CaNAT1* was cut with KpnI (for upstream homology) or SacI (for downstream homology). Primer design and cloning conditions were performed using NEBuilder^®^ HiFi DNA assembly master mix and NEBuilder^®^ Assembly tool (New England Biolabs, Ipswich, MA, USA), as per manufacturer’s protocol. Primers were designed to PCR amplify the homology arms (∼1000 bp) on either side of the target gene from genomic DNA isolated from *C. intermedia* strain CBS 141442. Amplified homology arms were cloned into the respective linearized plasmid resulting in two plasmids, each containing the same backbone but different homology arms.

### Transformation of *Escherichia coli*

All cloned plasmids were transformed into *E. coli* DH5α chemical competent cells (Sambrook and Russell 2006). Positive clones were identified using colony PCR, and propagated overnight in LB medium (1% tryptone, 1% NaCl and 0.5% yeast extract) supplemented with ampicillin (100 μg/ml) at 37°C and 1 × *g* followed by plasmid isolation using the GENEJet Plasmid miniprep kit (Thermo Fisher).

### Preparation of linear and split-marker DNA for yeast transformation

Prior to yeast transformation, plasmid p*CaNAT1* was linearized by restriction digestion with KpnI enzyme resulting in linearized plasmid, lin_*CaNAT1*.

Linearized cassette targeting the *ADE2* gene was PCR amplified using p*ADE2disr_CaNAT1* as template with primers annealing to the homologous arms. The resulting linear cassette contained *CaNAT1* marker flanked on either side by 1000 bp homologous arms to the *ADE2* gene in *C. intermedia*.

The split-marker fragments were generated using PCR such that the primers annealed to the homologous arms and in the markers, resulting in two fragments each containing up/downstream homology to the target gene fused to a part of the *CaNAT1* marker. Split-marker fragments had an overlap of 391 bp in the *CaNAT1* gene. Split-marker fragments derived from p*CaNAT1* and p*FLP_CaNAT1* are referred to as split*CaNAT1* and split*FLP_CaNAT1*, respectively.

### Yeast transformation

The transformation procedure was adapted from the protocol developed by De Backer et al. (1999) for the transformation of *C. albicans* by electroporation. In short, an overnight culture of *C. intermedia* CBS 141442 grown at 30°C and 1 × *g* shaking was used to start a culture at OD_600_ = 0.1 in 100 ml YPD (1% yeast extract, 2% bacto peptone, and 2% glucose). Once cells reached OD_600_ = 1, the culture was centrifuged (2600 × *g* for 3 min) and washed twice with ice-cold sterile deionized water and once with ice-cold 1 M sorbitol. Cells were centrifuged once again followed by resuspension in 160 μl of 1 M sorbitol. The resuspended cells were divided into 40 μl aliquots in microcentrifuge tubes. To each aliquot, 8 μl of DNA (with an optimal amount of 2 μg) was added. The transformation mix was transferred to a chilled electroporation cuvette (0.2 cm gap BioRad MicroPulser electroporation cuvettes, BioRad Laboratories, Hercules, CA, USA) and electroporated with a BioRad MicroPulser (BioRad Laboratories) at 1.5 kV. Cells were thereafter gently transferred to 1 ml of 1 M sorbitol in YPD and incubated at 30°C for 3 h with 1 × *g* of shaking. Cultures were spread on YPD agar (2%) plates with cloNAT (200 μg/ml) for selection and left to grow at 30°C for 48 h or until colonies emerged.

### Validation of yeast deletion mutant strains

For visual screening of *ade2* deletion strains, cloNAT resistant transformants were incubated at 4°C for 7–10 days on YPD agar plates and observed for pink/red colonies. For faster screening, transformants were replica plated onto agar plates with YNB medium (0.17% yeast nitrogen base, 0.5% of (NH_4_)_2_SO_4_, 2% glucose, and 0.079% complete synthetic medium) without adenine, on which *C. intermedia ade2* mutants could not grow but turned dark red within 24 h.

For screening of mutants without visible phenotypes, colony PCR was performed. Individual colonies of transformants were lysed in 15 μl deionized water and heated to 98°C for 10 min. PCR analysis was performed using PHIRE II polymerase (Thermo Fisher) with a primer pair designed to anneal upstream and downstream of the homologous flanking regions of the deletion cassette as well as a third control primer that anneal to the target gene. In this way, a successful PCR reaction resulted in either a band of ∼1200 bp identifying true deletion mutants, or a band of ∼1500 bp in false positive transformants where the target gene was still intact.

### Cell synchronization

Liquid cultures of *C. intermedia* CBS 141442 were prepared by inoculating 20 ml of YPD with fresh cells from YPD plates. Cultures were incubated overnight at 30°C with 1 × *g* of shaking. These precultures were then used to inoculate fresh cultures at OD_600_ = 0.2 and left to grow for 3 h at 30°C with 1 × *g* shaking. Cells were thereafter harvested and resuspended in 16 ml of YPD. To each culture, 4 ml of sterile solution containing deionized water and hydroxyurea was added to obtain the desired concentrations of hydroxyurea (0–200 mM). Cultures were incubated at 30°C with 1 × *g* of shaking and OD_600_ was measured over 3.5 h. Microscopy pictures were also taken at regular intervals using a Leica AF 6000 inverted microscope (Leica Microsystems, Wetzlar, Germany) and processed with the Leica Application Suite (LAS-X) software (version 3.4.2).

### Induction of FLP recombinase gene expression

Yeast carbon base (YCB) media (2.34%) with BSA (0.4%) was used to induce expression of the FLP recombinase gene from the integrated *FLP_CaNAT1* deletion cassette. Transformed colonies were cultured in YCB with BSA and grown over 96 h before being plated on YPD agar plates. After 48 h of growth, colonies were replica plated onto solid YPD containing cloNAT to check for regained antibiotic sensitivity due to FLP recombinase induction and activity.

## Results

### The *CaNAT1* dominant marker gene can be used for selection of *C. intermedia* transformants

**Figure 1.**
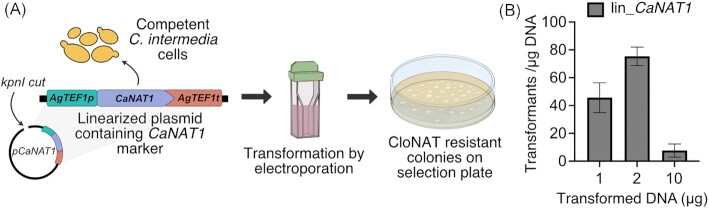
Transformation of *C. intermedia*. **(A)** Overview of the transformation protocol of *C. intermedia* using electroporation. Competent yeast cells were transformed with linearized plasmid (lin_*CaNAT1*), containing *CaNAT1* selection marker driven by *A. gossypii TEF1* promoter and terminator, resulting in cloNAT resistant colonies on selection plate. **(B)** Graph depicting the number of transformants obtained per µg of linearized plasmid DNA plotted against different amount of transformed DNA (µg) tested. Error bars represent standard deviation based on biological triplicates.

First, we set out to identify a working transformation protocol and a functional selectable marker gene for *C. intermedia*. Like many other CTG clade yeast (Vyas et al. 2015, Norton et al. 2017, Ennis et al. 2021, Strucko et al. 2021), *C. intermedia* displayed sensitivity to cloNAT in the concentration of 10–1000 µg/ml (data not shown), of which 200 µg/ml was chosen in further experiments. We transformed *C. intermedia* using electroporation and a linearized *CaNAT1* marker cassette that has proven effective in numerous yeast including *C. albicans, C. parapsilosis*, and *C. intermedia*’s close relative *Clavispora lusitaniae* (outlined in Fig. 1A) (De Backer et al. 1999, Shen et al. 2005). Transformations using 1, 2, and 10 µg of linearized plasmid DNA resulted in different number of transformants, where 2 µg DNA provided the highest number (average 78) of transformants per µg DNA (Fig. 1B) and was, therefore, used in subsequent transformations. A total of 16 randomly selected cloNAT resistant colonies all contained the *CaNAT1* cassette, as judged by the PCR products of expected size using primers that anneal to the *CaNAT1* gene (data not shown). As the plasmid DNA contained no significant homology to the *C. intermedia* genome, these results suggest that the DNA integrated randomly in the genome or possibly remained as linear or recircularized DNA in the nucleus. In either case, the experiment provides evidence that the transformation protocol resulted in the introduction and expression of the *CaNAT1* marker gene in *C. intermedia* cells, enabling selection of transformants.

To develop a protocol for targeted genome editing, we next created a cassette to disrupt the *ADE2* gene, where the *CaNAT1* cassette was flanked on both sides by 1000 bp of homology to the *ADE2* locus. In *ade2* mutants, phosphoribosyl aminoimidazole accumulates causing a red pigmentation of yeast cells (Dujon et al. 2004), which enables visual screening of the desired *ade2* mutants. Three rounds of transformations yielded a total of 274 cloNAT-resistant transformants, of which only two (<1%) turned red. PCR was used to confirm that the antibiotic-resistant, pink/red colonies had the *CaNAT1* marker integrated in the *ADE2* locus (data not shown). Although we have not investigated the mechanism(s) behind the DNA integrations observed, these results point to that *C. intermedia* preferentially uses NHEJ rather than HR to integrate foreign DNA in the genome, despite long homologous flanking regions of the deletion cassette.

### Cell synchronization increases the HR/NHEJ ratio in *C. intermedia*

**Figure 2.**
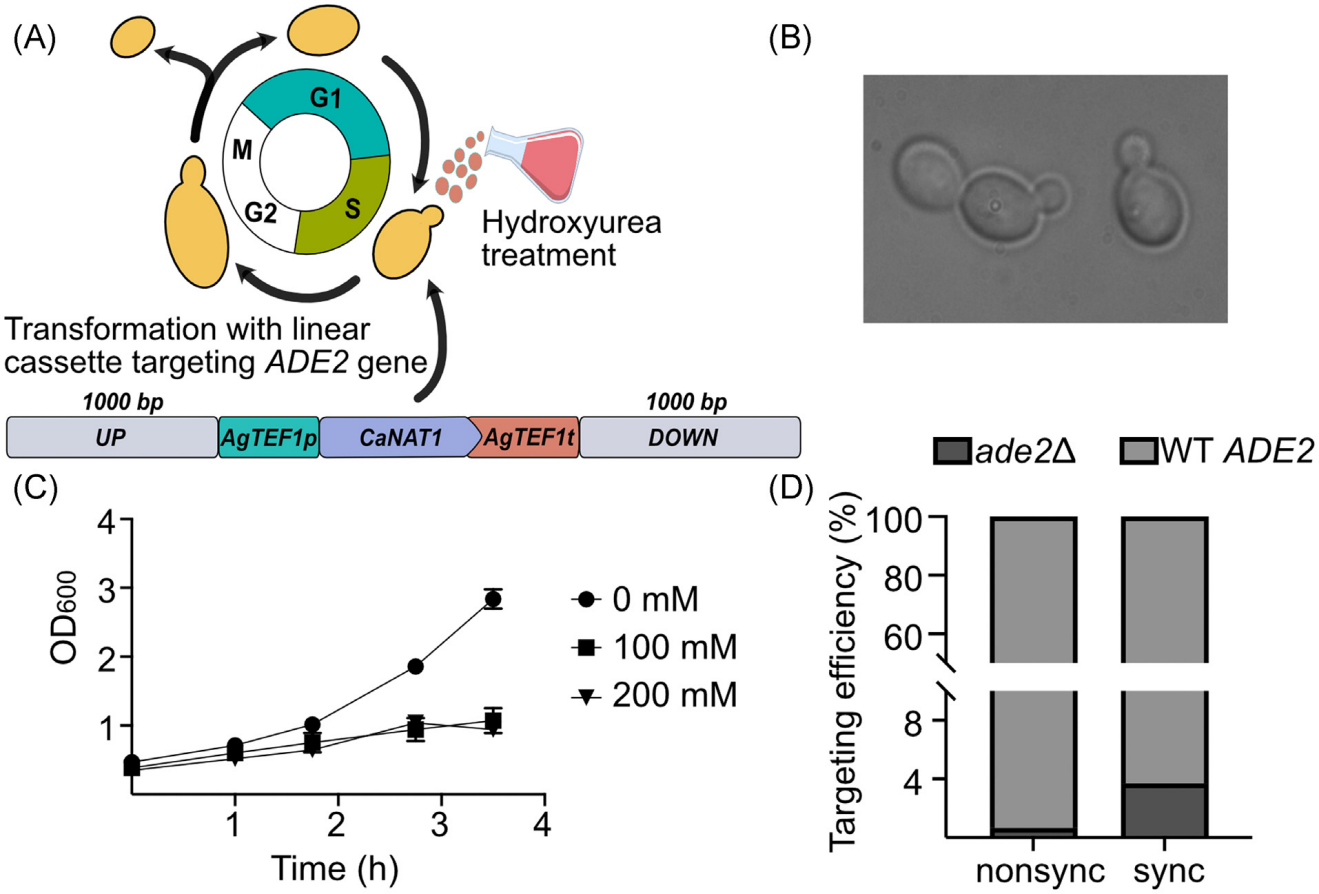
Cell synchronization using hydroxyurea. **(A)** Schematic representation of cell synchronization. Yeast cells arrested in S-phase of the cell cycle using hydroxyurea were transformed with lin_*ADE2disr_CaNAT1* cassette. **(B)** Photomicrograph showing cells arrested in their S-phase (also called the budding stage) after treatment with hydroxyurea. **(C)** Graphical representation of the change in biomass (OD_600_) over time (hours) of yeast cultures treated with different concentrations of hydroxyurea. **(D)** Targeting efficiencies achieved upon transformation of linear cassette, lin_*ADE2disr_CaNAT1*, targeting the *ADE2* gene in both nonsynchronized (nonsync) and synchronized (sync) cultures of *C. intermedia*. Legend shows targeted *ade2*deletion mutants (*ade2∆*) and *ADE2* transformants (WT *ADE2*). A break in the *y*-axis has been introduced to better visualize lower values for targeting efficiencies.

Having established a transformation protocol and a functional selection marker, we tried to increase the HR efficiency of *C. intermedia* by synchronizing cells in the S-phase using hydroxyurea (Fig. 2A). Both 100- and 200-mM concentrations of hydroxyurea were sufficient to block the cell cycle, as determined by microscopic examination (cells arrested in the large-budded stage) and optical density measurements (cease of biomass increase over time) (Fig. 2B and C). Synchronized cells (using 200 mM hydroxyurea) transformed with the linearized *ade2_CaNAT1* disruption cassette yielded 164 colonies, of which 3.7% proved to be pink/red *ade2* mutants. Thus, cell cycle synchronization resulted in a ∼5-fold improvement in HR efficiency compared to nonsynchronized cells (Fig. 2D).

### The split-marker approach has a large positive effect on gene targeting efficiency in *C. intermedia*

**Figure 3.**
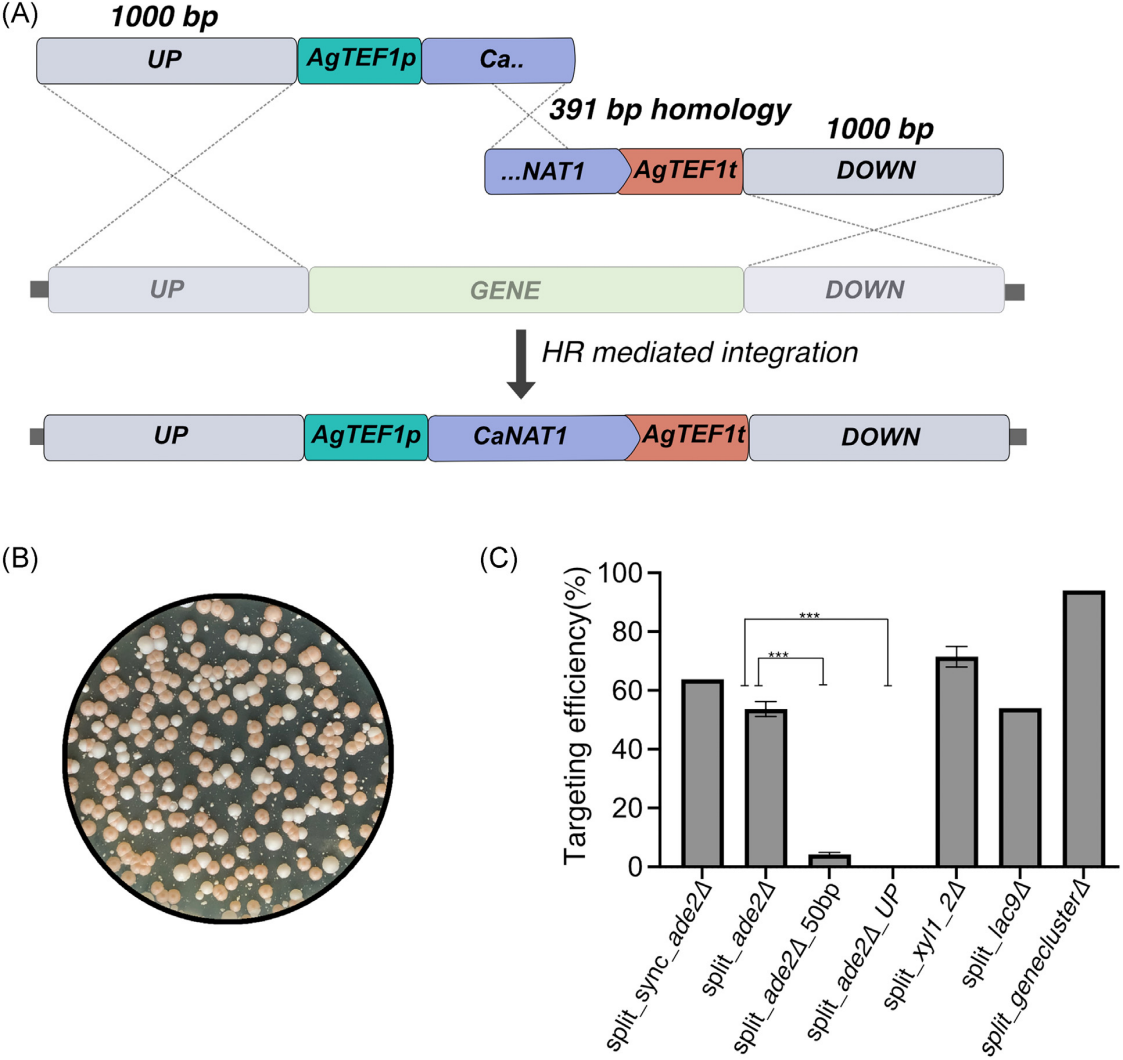
Split-marker method for genome editing in *C. intermedia*. **(A)** Schematic drawing of the integration of split-marker cassette at a target locus by HR. Split-marker cassettes contain either upstream/downstream (represented as UP/DOWN respectively) homology to the target gene and 391 bp homology at the *CaNAT1* marker. Homology between different fragments is represented by dotted lines. **(B)** A representative transformation plate displaying pink/red *ade2* mutants and white *ADE2* transformants growing on cloNAT selective medium. **(C)** Bar graph depicting targeting efficiencies using split-marker approach. Labels on *x-*axis: split-marker deletion with synchronized (split_sync*_ade2∆*), nonsynchronized cells (split_*ade2∆*), 50 bp homology split fragment targeting *ADE2* gene (split_*ade2∆_50 bp*), only one of the split fragments (split_*ade2∆_UP*), *XYLl_2* gene deletion (split_*xyl1_2*∆), *LAC9* deletion (split_*lac9∆*), and gene cluster deletion (split*_genecluster∆*) targeted in *C. intermedia*. Error bars represent standard deviation based on biological triplicates and statistical significance is determined using student’s *t*-test and is represented as “*” for *P* ≤ .05, “**” for *P* ≤ .01, and “***” for *P* ≤ .001.

To further improve gene deletion efficiency in *C. intermedia*, we thereafter employed the split-marker approach where the *CaNAT1* marker was divided into two partly overlapping fragments, each flanked by 1000 bp homologous target sequences (Fig. 3A). Both S-phase synchronized, and nonsynchronized cells were transformed with the split-marker fragments targeting the *ADE2* gene, to test the hypothesis that cells in the synchronized population would utilize the HR pathway to a larger extent than the nonsynchronized counterparts. Surprisingly, the HR efficiency increased to as much as 63.9% for synchronized and 55.7% for nonsynchronized populations, with the total number of *CaNAT1* resistant transformants being only marginally higher for the synchronized cells (119) than for nonsynchronized cells (115) (Fig. 3B and C). Thus, we can conclude that with our experimental setup, the split-marker approach had a greater positive impact on the HR/NHEJ ratio than cell synchronization. As cell synchronization extends the overall transformation time and hydroxyurea is classified as a toxic compound that must be handled with great care, we decided to skip this step in subsequent transformations.

As proof of concept, we used the split-marker approach to delete three additional loci in the *C. intermedia* genome. Targeting efficiencies were similar to *ADE2*: 71% for gene *XYL1_2* (gene size 948 bp), 67% for *LAC9* (2097 bp) encoding proteins with accession numbers SGZ56686 and SGZ49734, and 89% for a gene cluster of four genes (6577 bp) that is unique to *C. intermedia*, respectively (Fig. 3C). We speculate that the differences observed in targeting efficiency could be attributed to the genomic loci targeted, gene size and day to day experimental variation. We also assessed if short homology arms of 50 bp (split_ade2Δ_50 bp) were sufficient to direct the deletion cassette to the *ADE2* locus. However, only 3/78 (4%) transformants turned red, indicating the need for longer homology arms for efficient targeting using the split-marker approach (Fig. 3C). The split-marker approach repeatedly resulted in fewer transformants compared to linear deletion cassettes, and transformation of only the first of the two split-marker fragments (split_ade2Δ_UP) resulted in the absence of selectable transformants (Fig. 3C). Thus, transformants where HR between the marker gene fragments did not occur seem efficiently counter-selected for on the cloNAT plates. Overall, these experiments demonstrate the potential of the split-marker approach in improving HR efficiencies and enabling targeted gene deletions in *C. intermedia*.

### Marker recycling enables construction of double deletions in *C. intermedia*

**Figure 4.**
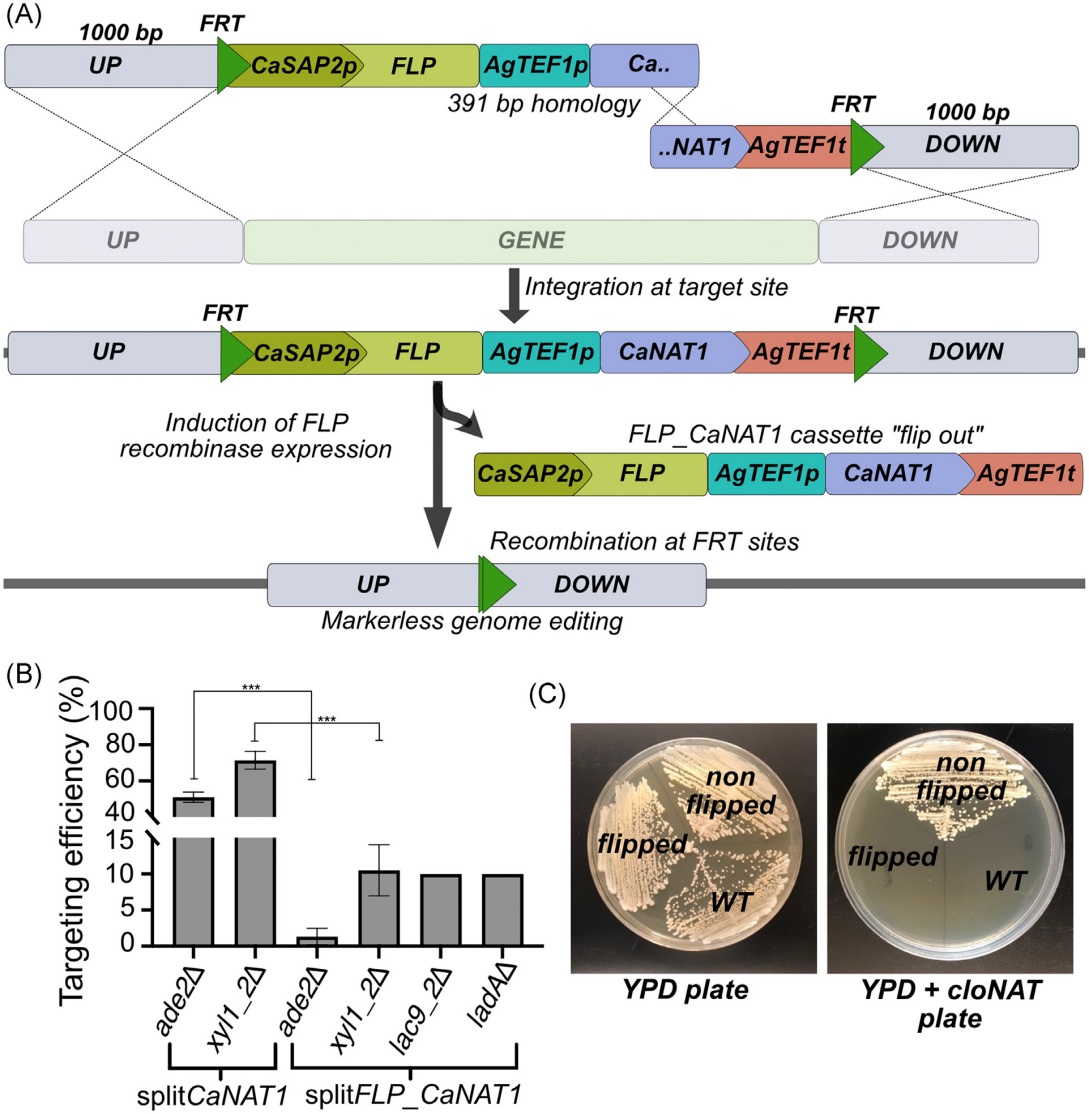
Gene deletion and marker recycling using *FLP* recombinase technique. **(A)** Schematic representation of HR-mediated integration of the split*FLP_CaNAT1* at target locus in *C. intermedia* genome followed by the induction of *FLP*-recombinase expression resulting in *FLP-CaNAT1* deletion cassette “flip out” and recombination of the FRT sites. Thus, the resulting transformant is marker free. **(B)** Targeting efficiencies using split*FLP-CaNAT1* compared to the split*CaNAT1* deletion cassette. A break in the *y*-axis has been introduced to better visualize lower values for targeting efficiencies. Error bars represent standard deviation based on biological triplicates and statistical significance is determined using student’s *t*-test and is represented as “*” for *P* ≤ .05, “**” for *P* ≤ .01, and “***” for *P* ≤ .001. **(C)** Plates depicting the selection for marker free transformants following removal of the *FLP-CaNAT1* cassette enabling marker recycling. Wild-type and a nonflipped colony are taken as controls for selection on cloNAT containing medium.

A drawback of the developed gene deletion protocol in *C. intermedia* is that the marker is integrated in the genome and cannot be recycled for subsequent deletions. To achieve a markerless engineered strain that can be used for additional genome editing or for industrial purposes where the microbial cell factories cannot contain antibiotic resistance markers, we combined the split-marker approach with a FLP recombinase. The split*FLP_CaNAT1* deletion cassettes were constructed using a p*FLP_CaNAT1* plasmid containing FLP recombinase gene in combination with the *CaNAT1* marker located between flippase recognition target (FRT) sites and flanked by a homologous region (Fig. 4A) (Shen et al. 2005). For easy comparison of the targeting efficiency, we deleted again *ADE2, XYL1_2* as well as genes *LAC9_2* and *LADA* (with protein accession numbers SGZ49734 and SGZ50177). Surprisingly, the targeting efficiency dropped to ∼10% for all tested loci (Fig. 4B). While further studies are needed to determine the cause of this marked decrease, one may hypothesize that the considerable larger size of the *FLP_CaNAT1* deletion cassette compared to the *CaNAT1* marker alone (3202 vs. 6121 bp) has a negative effect on the HR/NHEJ ratio (Štafa et al. 2017).

When applying the p*FLP_CaNAT1* plasmid in *C. albicans*, the *SAP2* promoter that drives the expression of the *FLP* gene in the deletion cassette is activated upon growth in medium with protein as the sole nitrogen source (Morschhauser et al. 1999). To test if the *CaSAP2* promoter functions in the same way in *C. intermedia*, and thereby can be used to flip out the *FLP_CaNAT1* cassette, the *C. intermedia* transformants were grown in medium containing BSA as protein source, streaked for single colonies on YPD plates without selection and thereafter transferred to cloNAT-containing plates. Like wild-type cells, transformants in which the deletion cassette had flipped could no longer grow on cloNAT-containing medium (Fig. 4C). In this way, we successfully constructed several transformants with marker-free deletions. However, it should be noted that we had to screen multiple colonies to identify cells where the *FLP_CaNAT1* cassette had flipped, and for some loci we even failed to flip out the cassette despite multiple attempts. This suggests that the *C. albicans SAP2* promoter or the FLP recombinase are not fully functional in *C. intermedia*. Nevertheless, we have managed to create a double deletion mutant by first deleting a gene cluster (the GAL gene cluster, 7027 bp) bp using the split*FLP_CaNAT1* cassette (targeting efficiency of 4%), followed by deletion of the *XYL1_2* gene using the split*CaNAT1* deletion cassette with a targeting efficiency of 60%. Although further optimization such as replacing the *CaSAP2* promoter with an endogenous inducible promoter may likely improve the overall efficiency of the process, our results clearly show that FLP recombinase technique in combination with split-marker cassette enable construction of double deletion mutants in *C. intermedia*.

## Discussion

The ability to manipulate chromosomally encoded genes within cells is a fundamental tool for dissecting the biology of an organism and to engineer microbial cell factories for bioproduction. In this study, we have developed a molecular toolbox for *C. intermedia*, including identification and optimization of a functional transformation protocol and deletion cassettes. Importantly, we improved the HR frequency from an original <1% to 55%–70%, enabling straight-forward targeted gene deletions and screening of transformants.

*De novo* development of genome-editing tools in new yeast species requires identification of functional transformation protocols and screening of cells that have taken up, integrated, and expressed the foreign DNA. Even though we benefitted greatly from previous work and tools developed for other CTG-clade yeast (De Backer et al. 1999, Morschhauser et al. 1999, Shen et al. 2005), each step of the process had to be tested and optimized for *C. intermedia*. Transformation of yeast is most often done using LiAc and heat shock or electroporation protocols (Gietz and Schiestl 2007, Kawai et al. 2010). In our hands, electroporation resulted in hundreds of *C. intermedia* transformants whereas the assessed LiAc-coupled heat shock protocol gave rise to 10-fold less colonies on the selection plates (unpublished results). The deletion cassettes employed in this study, in which the constitutive promoter *AgTEF1* drives the expression of selection marker *CaNAT1* that confers resistance to cloNAT seems to work in many CTG clade yeast (Shen et al. 2005, Norton et al. 2017, Strucko et al. 2021), and so also in *C. intermedia*. To identify additional markers, we tested also a CTG-compatible kanMX marker shown to function in several yeast (Hara et al. 2000, Millerioux et al. 2011). Unfortunately, transformation of the kanMX cassette did not result in any geneticin-resistant *C. intermedia* colonies. We also tried to generate uracil biosynthesis deficient strain by UV-mutagenesis followed by selection on 5-fluoroorotic acid-containing plates (Kohler and Fink 1996) but this experiment failed, as all screened colonies turned out to be auxotrophs for uracil (data not shown). This left us with only one selectable marker to work with and shows how difficult it can be to transfer knowledge and tools from one yeast to another.

Many yeast, including *C. intermedia*, display a high preference for ectopic integrations of foreign DNA molecules. In this study, we stepwise improved the HR frequency, where our first attempt to synchronize cells in the S-phase only helped to some degree (an increase from <1% to 3.7%). This method has proved efficient in other yeast and could possibly have been optimized further also for *C. intermedia*. However, the split-marker approach had a much larger positive effect on the HR/NHEJ ratio, as it increased efficiency by 50-fold as compared to linear DNA fragments. Such high HR efficiency using the split-marker approach circumvents the need to construct *ku70/80* mutants, which is both labor-intensive and limits molecular characterization in other strain backgrounds. It also avoids potential loss of fitness associated with NHEJ deficient mutants in some organisms, which may interfere with phenotyping of mutants in additional genes (Fraczek et al. 2018).

Unfortunately, the positive effect of the split-marker approach on HR frequency was largely lost when we applied it together with the *FLP-CaNAT1* cassette that enables marker-recycling and creation of marker-free transformants. Štafa et al. (2017) have shown that the size-ratio of transforming DNA to that of the homology arms can be a determining factor for the difference in targeting efficiency in *S. cerevisiae*. This might explain why we observed a significant difference in targeting efficiency when we switched from 1000 bp to 50 bp homology arms using the split*CaNAT1* cassette as well as from the split*CaNAT1* cassette (3200 bp in size) to the larger split*FLP-CaNAT1* cassette (6100 bp), both flanked by homology arms of 1000 bp on each side of the cassette. Optimization of this approach could, therefore, involve downsizing the cassette and possibly flanking it with even longer homology arms. Nevertheless, we successfully constructed double deletion mutants using the *FLP-CaNAT1* cassette and further use of this cassette could be to construct auxotrophic strains that can be used as a background in targeting other genes.

CRISPR/Cas9 technology holds great potential to revolutionize marker-free genome editing in nonconventional yeast, although development of these tools for new species can still be a highly challenging task. Besides having tools and protocols for transformation and selection, there is also a need to express functional and properly localized Cas9 proteins and sgRNAs, for which positive controls are often missing. Efficient CRISPR/Cas9-based genome editing is also limited by a low HR/NHEJ ratio in many species, forcing researchers to construct and screen for NHEJ-deficient strains. Using the split-marker approach for initial deletion of ku70 or ku80 can, therefore, pave the way for more efficient CRISRP/Cas9-based protocols, not limited to use in *C. intermedia*.

To summarize, in this study we have presented a simple gene deletion method based on the split-marker approach, which can be applicable for genome editing in virtually any yeast with preference for NHEJ over HR. Collectively, the results expand our knowledge on genome editing in yeast in general and *C. intermedia* in particular. The genetic tools developed herein will facilitate detailed molecular characterization of this yeast, both to decipher the genetics underlying *C. intermedia*’s capacity to metabolize different sugars and to develop it into an efficient cell factory for industrial use.

## Data Availability

Not applicable.
